# Profiling the gut and oral microbiota of hormone receptor-positive, HER2-negative metastatic breast cancer patients receiving pembrolizumab and eribulin

**DOI:** 10.20517/mrr.2024.49

**Published:** 2024-10-31

**Authors:** Nancy MY Teng, Andrea Malfettone, Matthew J Dalby, Raymond Kiu, David Seki, Tim Robinson, María Gion, Begoña Bermejo, José Manuel Pérez-García, Aleix Prat, Raúl Márquez Vázquez, Antonio Llombart-Cussac, Giuseppe Curigliano, Peter Schmid, Romualdo Barroso-Sousa, Mario Mancino, Eileen Shimizu, Jose Rodríguez-Morató, Leonardo Mina, Lindsay J Hall, Stephen D Robinson, Javier Cortés

**Affiliations:** ^1^Gut Microbes&Health, Quadram Institute Bioscience, Norwich NR4 7UQ, UK.; ^2^Medica Scientia Innovation Research (MEDSIR), Barcelona 08005, Spain.; ^3^Institute of Microbiology and Infection, University of Birmingham, Birmingham B12 2TT, UK.; ^4^Intestinal Microbiome, School of Life Sciences, ZIEL - Institute for Food&Health Technical University of Munich, Freising D-80333, Germany.; ^5^Bristol Medical School, University of Bristol, Bristol BS8 2BN, UK.; ^6^Medical Onology Department, Hospital Universitario Ramón y Cajal, Madrid 28034, Spain.; ^7^Medical Oncology, Hospital Clínico de Valencia, INCLIVA, CIBERONC, Medicine Department, Universidad de Valencia, Valencia 46010, Spain.; ^8^International Breast Cancer Center (IBCC), Pangaea Oncology, Quirón Group, Barcelona 08017, Spain.; ^9^Medical Oncology Department, Hospital Clínic y Provincial de Barcelona, Barcelona 08036, Spain.; ^10^Servicio de Oncología Médica, MD Anderson Cancer Center, Madrid 28033, Spain.; ^11^Department of medical oncology, Hospital Arnau de Vilanova, FISABIO, Valencia 46800, Spain.; ^12^European Institute of Oncology, IRCCS, University of Milano, Milano 20141, Italy.; ^13^Department of Oncology and Hemato-Oncology, University of Milano, Milano 20122, Italy.; ^14^Barts Cancer Institute, Queen Mary University of London, London E1 4NS, UK.; ^15^Oncology Center, Hospital Sirio-Libanes, Brasília-DF 70200-730, Brazil.; ^16^Norwich Medical School, University of East Anglia, Norwich NR4 7TJ, UK.; ^17^School of Biological Sciences, University of East Anglia, Norwich NR4 7TJ, UK.; ^18^Department of Medicine, Faculty of Biomedical and Health Sciences, Universidad Europea de Madrid, Madrid 28670, Spain.

**Keywords:** Microbiota, pembrolizumab, eribulin, immunotherapy, breast cancer, metastatic breast cancer, shotgun metagenomic sequencing, 16S rRNA gene amplicon sequencing

## Abstract

**Aim:** Changes in host-associated microbial communities (i.e., the microbiota) may modulate responses to checkpoint blockade immunotherapy. In the KELLY phase II study (NCT03222856), we previously demonstrated that pembrolizumab [anti-programmed cell death protein 1 (PD-1)] combined with eribulin (plus microtubule-targeting chemotherapy) showed encouraging antitumor activity in patients with hormone receptor (HR)-positive/human epidermal growth factor receptor 2 (HER2)-negative metastatic breast cancer (mBC) who had received prior treatments.

**Methods:** A total of 58 fecal and 67 saliva samples were prospectively collected from a subset of 28 patients at baseline (BL), after three treatment cycles, and end of treatment. Shotgun metagenomics, 16S rRNA gene amplicon sequencing, and bioinformatics and statistical approaches were used to characterize fecal and oral microbiota profiles.

**Results:** Treatment caused no substantial perturbations in gut or oral microbiota, suggesting minimal drug-related microbial toxicity. *Bacteroides* and *Faecalibacterium* were the dominant gut microbiota genera, while *Prevotella* and *Streptococcus* were present in both oral and gut samples, highlighting potential gut-oral microbial interactions. Additionally, clinical benefit (CB) appeared to be associated with gut-associated *Bacteroides fragilis* (*B. fragilis*) and a BL oral abundance of *Streptococcus* ≥ 30%. Notably, *B. fragilis* NCTC 9343 supernatant induced dose-dependent lactate dehydrogenase (LDH) release from the MCF-7 (HR-positive/HER2-negative) BC cell line.

**Conclusion:** These findings suggest that specific gut and oral microbiota may modulate the effectiveness of combinatory anti-BC therapies, potentially through the action of microbial metabolites.

## INTRODUCTION

Breast cancer (BC) is the most common cancer in women worldwide, with hormone receptor (HR)-positive (HR[+])/human epidermal growth factor receptor 2 (HER2)-negative (HER2[-]) BC being the most prevalent biological subtype^[1]^. Determining the optimal treatment for metastatic breast cancer (mBC) is complex and typically involves sequential therapies, often based on cytotoxic chemotherapy. These treatments carry an increased risk of cumulative toxicities, which are unique to each patient, and the potential for developing drug resistance.

Checkpoint inhibitor monotherapy has shown only modest activity in HR[+]/HER2[-] mBC^[2]^. However, the addition of pembrolizumab to standard neoadjuvant therapy was found to significantly improve the pathological complete response rate in HER2[-] early BC patients^[3]^. The KELLY trial (NCT03222856) evaluated the safety and efficacy of combining pembrolizumab with eribulin for the treatment of HR[+]/HER2[-] mBC. The KELLY trial demonstrated a clinical benefit (CB) rate of 56.8% [95%CI (confidence interval): 41.0%-71.7%] and a median progression-free survival (PFS) of 6 months (95%CI: 3.7-8.4 months)^[4]^, exceeding the activity observed with eribulin monotherapy in a similar patient population^[5]^.

Factors predicting responses to immunotherapy may be related to either the patient or the tumor. Over the past decade, numerous studies have explored the relationship between the gut microbiota and clinical outcomes in anti-cancer therapies^[6-11]^. Notably, higher gut diversity has been associated with better responses to anti-programmed cell death protein 1 (PD-1) immunotherapy in metastatic melanoma patients^[11]^, and *Bifidobacterium* species and strains have been shown to enhance antitumor activity in anti-programmed cell death ligand 1 (PD-L1) therapies^[6,7,12]^. These changes in gut microbiota composition are thought to modulate immune response, potentially enhancing T-cell activity through the production of specific microbial metabolites^[7,11,13-15]^.

While most studies have focussed on strongly immunogenic cancers such as melanoma, few have explored microbiota profiles in poorly immunogenic cancers such as BC^[16]^. Additionally, the majority of these studies focus on the gut microbiota’s correlation with anti-cancer therapies, with limited attention given to the oral microbiota. Emerging evidence suggests that bacterial dissemination between the oral and gut microbiota may disrupt the gut microbiota ecosystem, potentially leading to adverse health outcomes^[17]^.

The CALADRIO study was an exploratory retrospective analysis aimed at evaluating the associations between both the oral and gut microbiota and clinical outcomes of patients included in the KELLY study.

## METHODS

### Study design and sample collection

The KELLY study (NCT03222856) was a phase II clinical trial assessing the safety and efficacy of a novel combination therapy of pembrolizumab and eribulin for HR[+]/HER2[-] mBC patients^[4]^. Eligible patients were ≥ 18 years old and had previously received hormone therapy and one to two lines of chemotherapy for advanced disease. The primary endpoint of the KELLY study was to determine the CB rate, defined as the percentage of patients experiencing a complete response, partial response or stable disease enduring equal to or more than 24 months, according to the Response Evaluation Criteria in Solid Tumors (RECIST) v1.1^[4]^.

The CALADRIO study (NCT03222856, EUDRACT: 2016-004513-27) presented here is an exploratory retrospective analysis aiming to profile the oral and gut microbiota of a subset of patients enrolled in the KELLY study to establish if microbiota-associated profiles could be used as predictive biomarkers of responses and/or CB. Consenting patients from the KELLY study (*n* = 28) provided fecal and saliva samples at baseline (BL), after three treatment cycles [Cycle 4 Day 1, (C4D1)], and at end-of-treatment (EoT). A total of 65 fecal samples and 70 buccal samples were collected. Specifically, fecal samples collected were: BL 28/28, at C4D1 22/22, and at EoT 15/20, and all buccal samples were collected at all time points. Fecal samples were collected as stipulated by the International Human Microbiome Standard guidelines: SOP 03 V1^[18]^, and stored in a deoxyribonucleic acid (DNA) preservation buffer.

All patients included in the study provided written informed consent (including biomarker analyses on biological samples) before any protocol-related activities started. The study protocol, patient information leaflet, and informed consent form were reviewed by each location’s Ethics Committee. The study was conducted in accordance with the Declaration of Helsinki, the International Council for Harmonization of Technical Requirements for Pharmaceuticals for Human Use Good Clinical Practice guidelines, and all applicable regulations and laws.

### Sample processing and sequencing

Saliva samples (5 mL each) were collected from all patients into sterile containers, aliquoted, and stored at -80 °C until further processing. A total of 2 mL of each saliva sample was centrifuged for 15 min at 2,600 g. The pellet was resuspended with 750 μL of the PowerSoil bead solution. Then, the sample was loaded into the PowerLizer® Glass Bead Tube and the bacterial DNA was extracted using QIAamp® PowerFecal Kit (Qiagen, 51804) according to the manufacturer’s instructions. 16S ribosomal ribonucleic acid (rRNA) gene amplicon sequencing was performed as previously described^[19]^ for a total of 70 samples. Three oral samples were removed from the downstream analysis as we did not obtain the PD-L1 status of one patient who provided two samples and one patient withdrew consent.

Fecal samples were shipped to the Quadram Institute Bioscience, Norwich, United Kingdom, for processing. Processing and quantification of the fecal samples were done using the Promega Maxwell RSC PureFood GMO and Authentication kit (Promega, AS1600). A 200 µL aliquot of fecal sample was transferred into a Lysing Matrix E tube. Then, 1 mL of CTAB was added to the sample and vortexed for 30 s before being incubated on a heat block at 95 °C for 5 min, and then vortexed for a minute. Samples were homogenized in a FastPrep-24 machine for 45 s at a speed of 6.0 m/s. To each sample, 40 µL of Proteinase K and 20 µL of RNase A were added and vortexed to mix. The samples were heated to 70 °C for 10 min, while cartridges were prepared for the Maxwell robot according to the manufacturer’s instructions. DNA was quantified using Qubit 2.0 dsDNA BR assay kit (ThermoFisher Scientific, Q32850). For fecal samples, library preparation was done following the manufacturer’s instructions using the DNA flex kit with 8 mer UDI and then sent to Source Biosciences (Cambridge, United Kingdom) to be sequenced on the Novoseq platform, 2 × 250 bp up to 750 Gb. A total of 65 fecal samples were submitted for shotgun metagenomic sequencing. Four samples were below the minimum quality for downstream analysis and three samples were removed. One patient withdrew consent. We removed two other samples because the patient’s PD-L1 status could not be assessed due to low neoplastic cellularity.

For shotgun sequencing (fecal samples only), the mean read depth was 4.14 Gbp, the median number of reads of this dataset was 30,197,291 reads, and the mean number of reads per sample was 27,592,595. For 16S rRNA gene amplicon sequencing (saliva samples), the mean read depth was 37.2 Mbp, the median number of reads in this dataset was 122,973 reads, and the mean number of reads per sample was 12,378.

### Bioinformatics and data analysis

16S rRNA gene amplicon sequencing assembly was done using Qiime v1.9.1 as previously described^[20]^. Once operational taxonomical units (OTUs) were assigned, biological observational matrix files were generated and data visualized on MEGAN v6.20.19^[21]^ or on R^[22]^ with the package “Phyloseq-v3.18”, and samples with less than 20,000 reads were removed. Data were normalized using variance stabilization with DeSeq2 to relative abundances (%)^[23]^.

For metagenome shotgun sequencing, fastp v0.20.0^[24]^ was used to run a quality filtering on the FASTQ reads with a -q 20. KneadData v0.10.0^[25]^ was used to remove host-associated reads, i.e., human reads, using the database “GrCh38_noalt_decoy_as” using --bypass-trim, --reorder and --bypass-trf (found at: https://benlangmead.github.io/aws-indexes/bowtie). Metagenome sequences were co-assembled using Megahit v1.2.9^[26]^, run via MetaWrap v1.3.2^[27]^. To assign taxonomy, Kraken2 v2.1.2^[28]^ was used with a confidence level of 0.1 on the FASTQ reads and the output run through Bracken v2.6.2^[29]^ to accurately estimate relative abundances with a threshold of 10. Metagenome assembled genomes (MAGs) were computationally extracted via MetaWrap v1.3.2 (Concoct v1.1.0^[30-32]^, Maxbin2^[33]^, and MetaBAT2^[34]^) based on metagenome sequencing reads and co-assemblies. Bin refinement was performed and only high-quality MAGs with the parameters > 80% completeness and < 10% contamination (via CheckM v1.1.3) were used for further analysis. GTDB-tk v 1.5.1^[35]^ was utilized to assign taxonomy to the MAGs extracted at the species level.

For functional assignment, we used Humann3 v3.0.0^[25]^ via Metaphlan v3.0.13^[25]^ pipeline, using Chocophlan (downloaded 24 August 2021) as the database. The output file “humann_all_pathabundance_cpm.tsv” was normalized and then stratified before data analysis.

Data analysis was performed using RStudio 2022.07.1+554 “Spotted Wakerobin” Release and R version 4.2.2^[22]^. Alpha diversity was assessed by Shannon diversity index, while beta diversity was evaluated using non-metric distance scaling (NMDS) with the package “Vegan- 2.6-4”. Statistical significance of alpha diversity was performed using Kruskal-Wallis, while beta diversity was assessed with PERMANOVA using adonis2 as part of the “Vegan package v2.6-4”^[36]^. Linear discriminant analysis effect size (LEfSe) analysis followed the statistical analysis described in^[37]^. Plots and figures were created using “ggplot2-v3.4.3”. A *P* < 0.05 was taken to be significant. The statistical tests performed are specified in the figure legend.

To calculate the clustering for the heatmap, we used Bray-Curtis dissimilarity matrix for the vegdist function and average for the hclust function, i.e., sample and genus clustering, and hierarchal clustering, respectively. We used the Huttenhower LEfSe^[37]^ Galaxy pipeline. For *Bacteroides fragilis* (*B. fragilis*) MAG screening, we used the METABOLIC pipeline as described in^[38]^ and utilized Resfinder^[39,40]^ to assess antibiotic resistance potential. Using the paper by Franco^[41]^, we downloaded 22 genomic sequences to run through Abricate v1.0.1^[42]^ and screen for the presence or absence of the *bft* gene [Table 1].

**Table 1:** NCBI Accession numbers of the 22 genomic sequences for the *bft* gene

**Accession number (Genbank)**	**Genome location (bp-bp), (c, chromosome)**
AB026626.1	345-1538
CP098482.1	2670205-2671398
CP098482.1	c5312584-5311391
JAHYPF010000020.1	55345-56538
JALFMY010000010.1	81936-83129
JANUSS010000001.1	3806129-3807322
JANUTD010000001.1	2513275-2514468
JANUTH010000001.1	2513065-2514258
JANUTZ010000001.1	347095-348288
JANUTZ010000002.1	1981850-1983043
JANUUF010000001.1	2527119-2528312
JANUUF010000001.1	347004-348197
NZ_CP011073.1	4564579-4565772
NZ_CP098482.1	c5312584-5311391
NZ_JAPUAE010000018.1	56787-57980
NZ_JGEF01000023.1	64376-65569
NZ_JH724206.1	c3476585-3475392
NZ_JH724218.1	c72677-71484
NZ_LIDS01000027.1	55915-57108
NZ_LIDT01000031.1	c8881-7688
NZ_LIDV01000087.1	c315654-314461
NZ_PDCT01000007.1	c60762-59569

### *In vitro* testing of *B. fragilis* supernatants with BC cell lines

To obtain soluble *B. fragilis* metabolites for testing *in vitro*, we cultured the type strain *B. fragilis* NCTC 9343 anaerobically (85%/5%/10% for N_2_/CO_2_/H_2_ respectively) at 37 °C. The supernatant of the cultures was harvested at exponential [14 h, optical density (OD) 1.01], late exponential (18 h, OD 1.13), stationary (26 h, OD 1.11), and death phase (36 h, OD 1.04) of growth and filter-sterilized (0.22 µm). Sterile aliquots were kept at -20 °C until further use. MCF-7 BC cells were seeded at a density of 9,000 cells per well in a 48-well plate in complete media (DMEM high-glucose, no phenol red, supplemented with 5% fetal bovine serum, 1% L-glutamine, 1% Pen/Strep, 1% sodium pyruvate). The cells were exposed to the cell-free bacterial supernatant (CFS) at 1:5 dilution for various time points (2 h up to 36 h) before being replaced with minimal media (DMEM high-glucose, no phenol red, supplemented with 2% fetal bovine serum, 1% L-glutamine, 1% Pen/Strep, 1% sodium pyruvate) with 10% MTS (Abcam, ab197010) to measure cell viability. The reagent was left to incubate for 1 h before reading at OD_490_ nm. Controls included: cells in minimal media, cells in complete media, lysed cells as a positive control for lactate dehydrogenase (LDH) assay, and brain heart infusion (BHI) with cell media as the background control. A LDH assay (Promega, G1780) was performed with the CFS after initial incubation, according to the manufacturer’s instructions.

## RESULTS

### Patient characteristics and summary of sequencing data

A total of 28 patients provided clinical samples for the CALADRIO study, with 16 patients experiencing a CB (57%, 95%CI: 38.8%-75.4%). The median age of patients was 53.5 years (range: 47.5-63.0 years) and 48.1% of patients had PD-L1 positive tumors (combined positive score ≥ 1 using the 22C3 pharmDx assay), as shown in Table 2^[4]^. We collected 65 fecal and 70 buccal samples: BL 28/28, C4D1 22/22, and EoT 15/20. Four fecal samples were excluded due to insufficient sequencing depth and an additional three samples were removed due to missing clinical data or withdrawn consent. In total, 58 fecal samples and 67 saliva samples were used for final analysis.

**Table 2:** Patient BL characteristics and samples collected

**Characteristic**	**Overall (*n* = 28)**
Female (%): age, median (IQR), years	100: 53.5 (47.5-63.0)
ER status (%): positive | negative	100 | 0
PgR status (%): positive | negative	82.1 | 17.9
PD-L1 status (%)^a^: positive | negative	48.1 | 51.9
Primary endpoint: CB | no-CB [*n*, (%)]	16 (57%) | 12 (43%)
Samples collected (*n*): fecal | saliva	65 | 70

Data are %, unless otherwise indicated. ^a^PD-L1 status was not evaluable in 1 patient because of low tumor cellularity. PD-L1 protein expression was determined by using CPS, which is the number of PD-L1 staining cells (tumor cells, lymphocytes, macrophages) divided by the total number of viable tumor cells, multiplied by 100. The specimen was considered to have PD-L1 expression if CPS ≥ 1 according to the PD-L1 IHC 22C3 pharmDx assay. BL: Baseline; IQR: interquartile range; ER: oestrogen receptor; PgR: progesterone receptor; PD-L1: programmed death-1 ligand 1; CB: clinical benefit; CPS: combined positive score.

### The combination therapy of P + E did not confer microbiota toxicity

Microbial alpha diversity was assessed at each sample collection time point using Shannon’s diversity index to evaluate whether treatment impacted overall microbial diversity. No significant drug-related changes were observed in either gut or oral microbiota alpha diversity, which was also confirmed at the genus level by Kruskal-Wallis test, *P* = 0.43 (gut) and 0.72 (oral).

When comparing overall alpha diversity, there were no substantial differences at the genus level between CB and no-CB groups for both the gut [Supplementary Figure 1A] and oral [Supplementary Figure 1B] microbiota profiles across all time points. However, we observed that Shannon diversity remained stable in the CB group, while diversity fluctuated in the no-CB group, decreasing in the gut and increasing in the oral profiles.

No significant differences were found in beta diversity after treatment with pembrolizumab and eribulin. Specifically, there was no clear segregation of either gut or oral microbiota profiles based on clinical metadata [PD-L1 status, neutrophil-to-lymphocyte ratio (NLR, with CB and without CB), CB status or PFS (> 6 months and ≤ 6 months)]. For the gut microbiota, this was confirmed using PERMANOVA analysis: *P* = 0.23, 0.66, 0.27, and 0.05 at the genus level and *P* = 0.44, 0.20, 0.38, and 0.14 at the species level for PD-L1, NLR (a reliable marker of immune response), CB status, and PFS, respectively. Similarly, for the oral microbiota at the genus level, *P* = 0.09, 0.45, 0.26 and 0.50 for PD-L1, NLR, CB status, and PFS, respectively (data not shown). Therefore, we conclude that the combination therapy of pembrolizumab and eribulin does not confer significant microbiota-related toxicities.

### Shared taxa between the oral and gut microbiota

**Figure 1:**
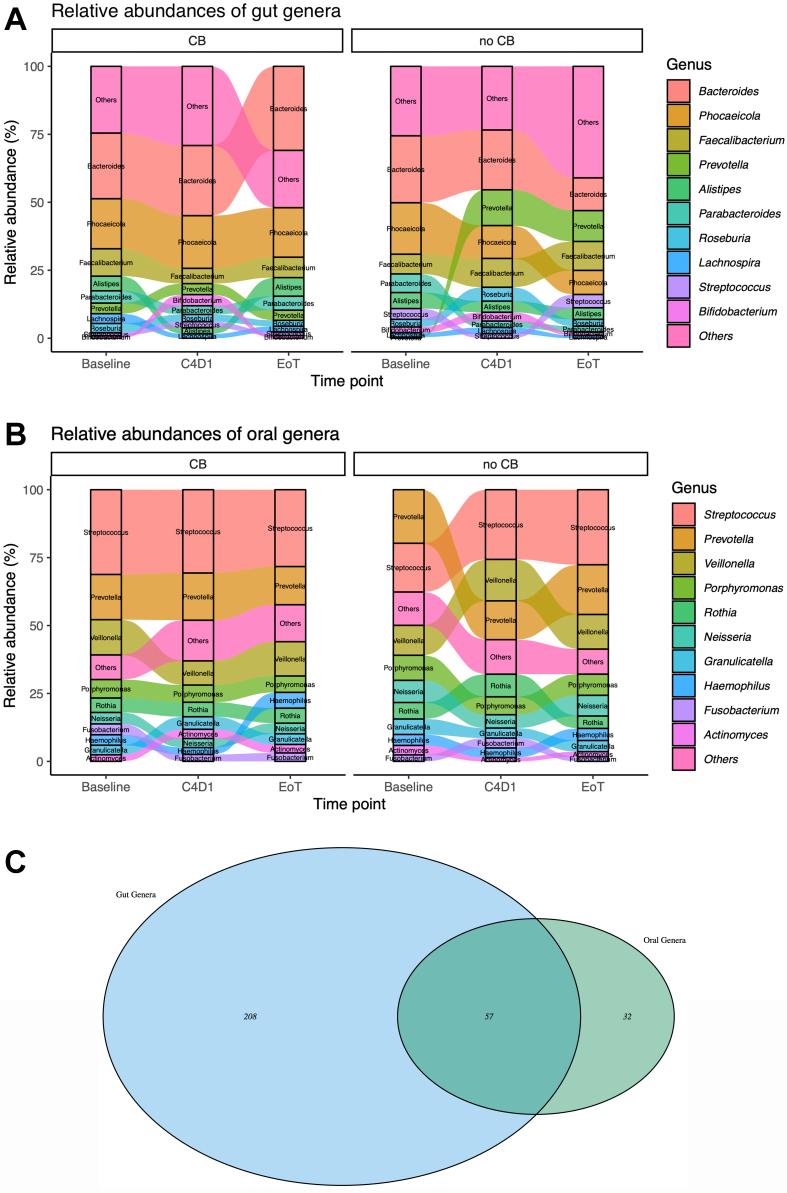
Relative abundances of (A) gut and (B) oral microbiota profiles showed little changes throughout the study treatment, thereby indicating that pembrolizumab and eribulin do not confer microbiota toxicity; (C) There were 57 common genera between the oral and gut microbiota profiles.

We assessed specific taxonomic profiles using alluvial and stacked bar plots of the top ten genera to visualize temporal changes in taxa, stratified by clinical parameters. Although moderate changes in *Bacteroides* were across time points, these were not statically significant and may have been influenced by the treatment or unaccounted factors such as diet. Changes were also noted in gut-associated *Prevotella* [Figure 1A], though further analysis indicated that this expansion was driven by three patients who had > 20% *Prevotella* in their profiles. In the oral microbiota profiles [Figure 1B], a reduction in *Prevotella* was observed in the no-CB group, although these samples were not paired with those from the same patient showing changes in the gut.

Overall, no substantive differences were detected in either the oral or gut microbiota based on clinical parameters, but 57 genera were common to both oral and fecal samples [Figure 1C]. This overlap may suggest potential oral-gut translocation, though further sequencing at greater depth is necessary to explore this possibility in more detail.

### Potential functions of the gut microbiota in CB and no-CB patients are similar

**Figure 2:**
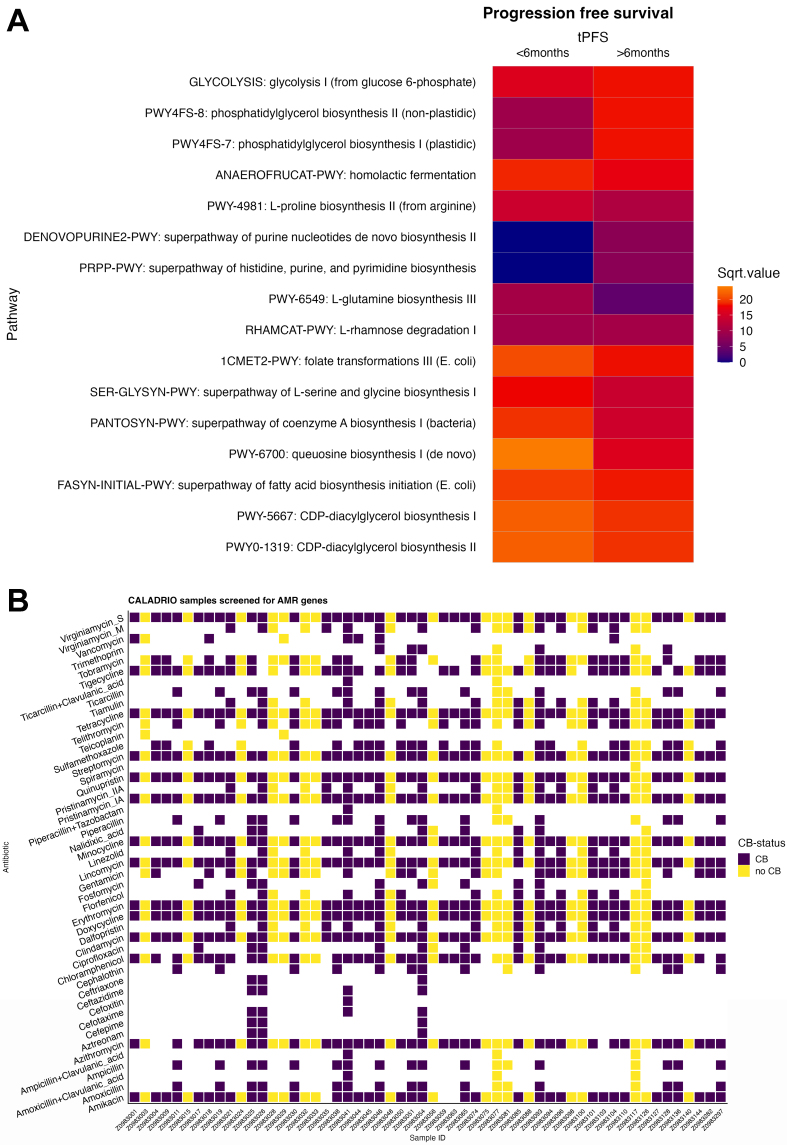
(A) Heatmap of metabolic pathways identified to have a LDA > 2.0 using LEfSe analysis for the clinical feature: time to PFS. Unstratified abundances of all pathways produced by Humann3 were run through LEfSe. Pathways identified as a discriminative feature as a LDA > 2.0 was then selected to create the heatmap. We categorized PFS into two groups: > 6 months and ≤ 6 months; (B) Antibiotic resistance genes identified in CALADRIO samples using Resfinder associated with CB. LDA: Linear discriminant analysis; LEfSe: linear discriminant analysis effect size; PFS: progression-free survival; CB: clinical benefit.

The overall functional potential^[25]^ of gut microbiota samples was assessed using shotgun metagenomic sequencing. Consistent with the taxonomic profiles, no significant differences were observed between CB and no-CB groups. However, LEfSe analysis identified two functional pathways that were significantly associated (*P* < 0.05) with the clinical parameter PFS. These pathways were: “DeNovo Purine2 Pathway” and “PRPP Pathway” [Figure 2A]. The DeNovo Purine 2 pathway is involved in purine nucleotides *de novo* biosynthesis II, while the PRPP Pathway is related to histidine, purine, and pyrimidine biosynthesis.

When analyzing antibiotic resistance genes within the gut microbiota, no distinct pattern was observed between CB and no-CB groups [Figure 2B]. Given previous findings that cytochrome (CYP) P450 isoform 3A4 can metabolize eribulin^[43-45]^, we also examined microbial CYP genes in relation to CB status. Interestingly, patients who experienced CB exhibited a higher abundance of *CYP* genes in their gut microbiota [Supplementary Figure 2].

### Oral *Streptococcus* and gut *B. fragilis* as potential biomarkers for CB

**Figure 3:**
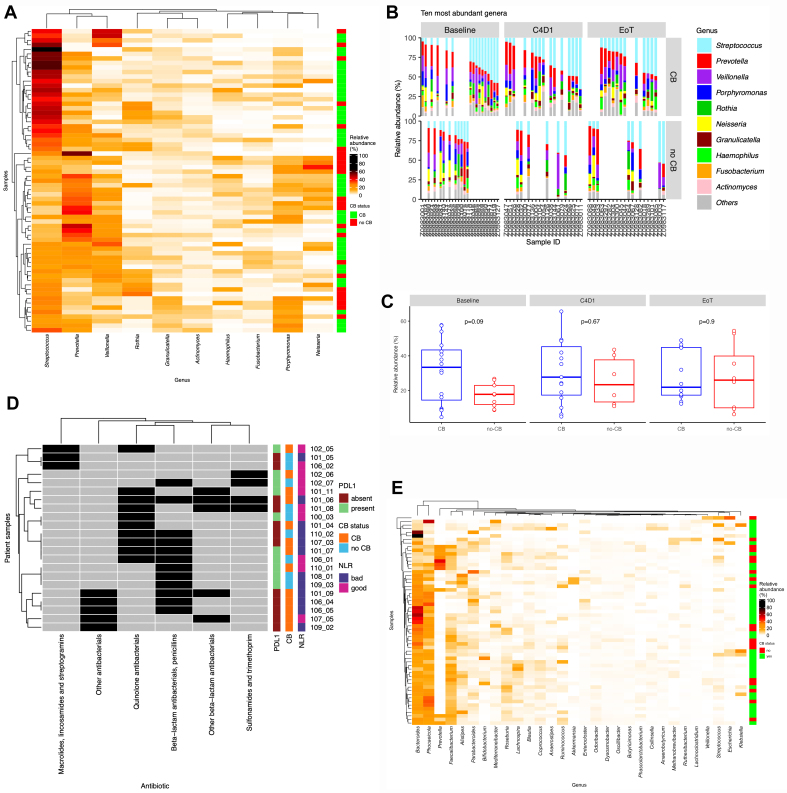
Samples with a relative abundance of > 30% Streptococcus came from patients who experienced a CB. (A) Heatmap by Bray-Curtis dissimilarity matrix on saliva samples by the top ten genera; (B) further visualization showed this tended to be BL samples. This difference between oral Streptococcus in CB and no-CB group was lost at consecutive time points (C), most likely due to a combination of antibiotic usage and a lower number of EOT samples provided. Mann-Whitney U test was performed on the CB *vs.* no-CB group where no significance was observed. Antibiotics, categorized by class, taken by patients based on clinical metadata. Not all patients enrolled in the CALADRIO study had complete metadata about antibiotics, which is why the number of patients presented differs from the number of patients enrolled (D). No clustering of gut microbiota data was observed according to CB status, suggesting no overall taxonomic changes (E). Mann-Whitney U was performed to compare the relative abundances of Streptococcus by time point. CB: Clinical benefit; BL: baseline; EOT: end-of-treatment.

While diversity and overall taxonomic profiles did not reveal significant differences, more nuanced changes in specific microbiota members may occur during treatment. Heatmap visualization of the oral microbiota, clustered using a Bray Curtis dissimilarity matrix, suggested that patient samples with a relative abundance of > 30% *Streptococcus* were associated with CB [Figure 3A]. The trend was most evident at BL [Figure 3B], where patients with a CB appeared to have slightly higher relative abundances of *Streptococcus*, although this finding was not statistically significant [Figure 3C]. No significant differences were observed at subsequent time points (C4D1 and EoT), possibly due to the effects of antibiotic administration [Figure 3D]. A similar analysis of the gut microbiota data showed no correlation with CB status [Figure 3E].

**Figure 4:**
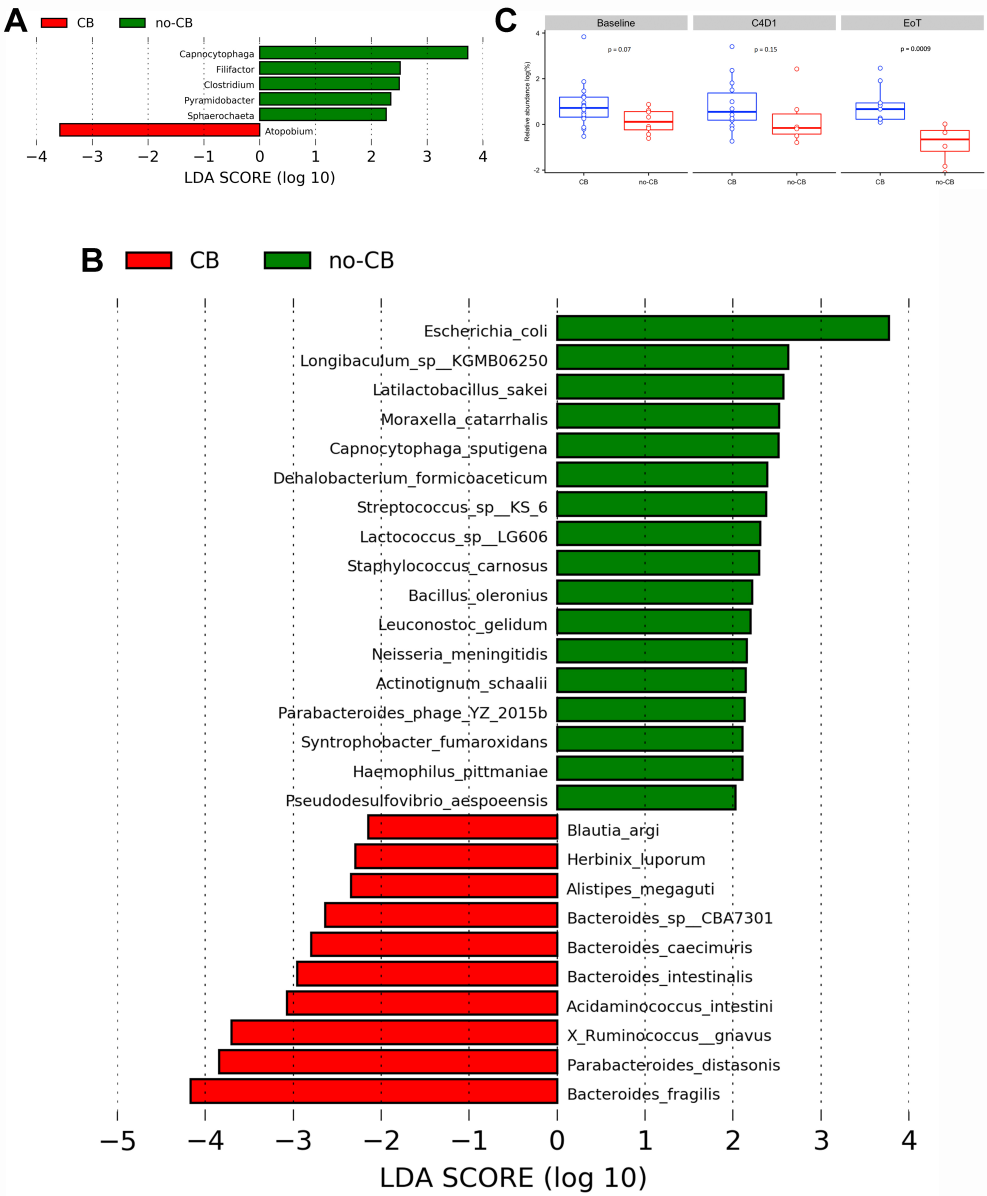
LEfSe analysis of the salivary microbiota data showed five genera associated with no-CB and one for CB (A). *B. fragilis* in the gut is associated with CB. LEfSe analysis (B) showed that *B. fragilis* is associated with CB. Box plots (C) further showed that patients with CB consistently had a greater relative abundance of *B. fragilis* than no-CB. This observation was most significant at EoT [Mann Whitney U: *P* = 0.009, U = 0, median (yes, no) = 1.95, 0.38, range (0.00-11.73)]. LEfSe: Linear discriminant analysis effect size; CB: clinical benefit.

LEfSe was applied to identify genera and/or species that characterize the CB and no-CB groups. In the oral microbiota, LEfSe analysis revealed six genera with discriminative features: *Capnocytophaga*, *Filifactor*, *Clostridium*, *Pyramidobacter*, and *Sphaerochaeta* were associated with no-CB, while only *Atopobium* was associated with CB [Figure 4A]. *Capnocytophaga*, *Filifactor*, and *Clostridium* were also detected in gut samples, but only *Capnocytophaga* showed an association with no-CB.

LEfSE analysis of gut microbiota identified 30 potentially significant taxa correlating with CB status [Figure 4B]. Notably, *B. fragilis* was enriched in the CB group, while *Escherichia coli* was associated with no-CB. Given previous research suggesting *B. fragilis* may act as a key mediator of antitumor effects in cancer immunotherapy and serve as a promising therapeutic target^[14,46,47]^, further analysis was undertaken. Visualization showed an increasing trend in the relative abundance of *B. fragilis* in the CB patients compared to the no-CB group [Figure 4C], with a statistically significance difference observed at EoT (*P* = 0.0009).

### Screening functional potential of *B. fragilis*

Given the therapeutic potential of *B. fragilis*, we performed a genomic analysis of this species using shotgun metagenomics data^[14]^. Five *B. fragilis* MAGs were extracted, most closely matching the type-strain *B. fragilis* NCTC 9343, with 4 out of 5 MAGs aligning at > 98%. Notably, all MAGs, except Z0983077.bin.48, originated from patients who experienced a CB (data not shown).

**Figure 5:**
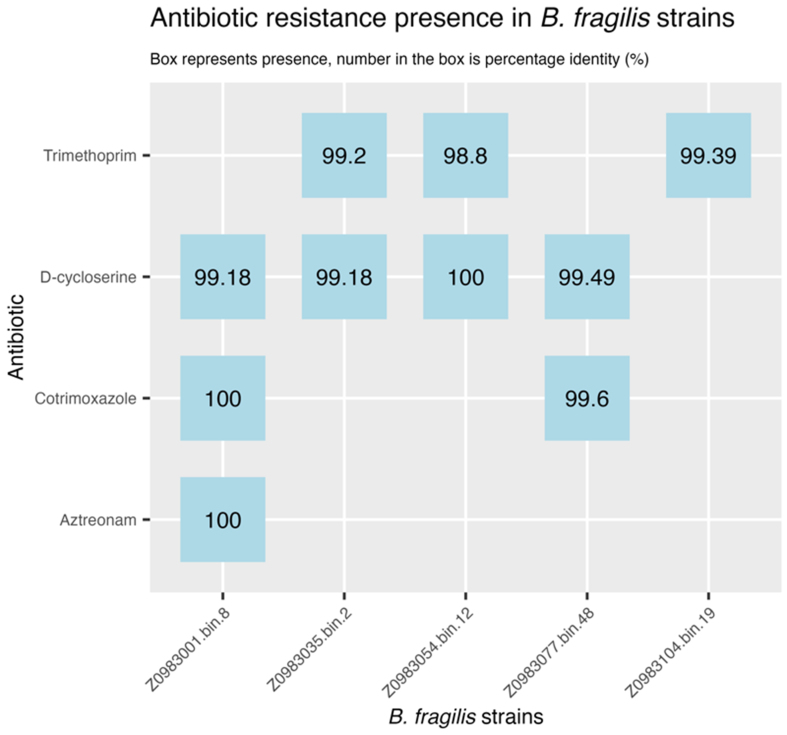
Antibiotic resistance presence in *B. fragilis* MAGs based on ResFinderFG. MAGs: Metagenome assembled genome.

To investigate the functional potential of *B. fragilis* in CB patients, we screened the MAGs for antimicrobial resistance determinants. Genes conferring resistance to trimethoprim, tetracycline, D-cycloserine, cotrimoxazole, and aztreonam were identified [Figure 5]. Among these, trimethoprim was the only antibiotic administered to the patients, suggesting that antibiotic resistance alone does not explain the higher relative abundance of *B. fragilis* in CB patients.

Further analysis using the METABOLIC pipeline indicated a specific cluster of sulfur cycling enzymes, i.e., methionine metabolizing enzymes^[48]^, present in these MAGs. Additionally, we screened for immunomodulatory components, focusing on capsular polysaccharide and the *B. fragilis* toxin (BFT), as these components are known to influence immune responses^[47]^. Three out of the five *B. fragilis* MAGs contained a putative protein involved in capsular polysaccharide export (Genbank: BAD47972.1)^[49]^, while none of the MAGs encoded the *bft* gene (fragilysin), as summarized in Table 1.

### *B. fragilis* conditioned media induces intracellular LDH release in an HR[+]/HER2[-] BC cell line

Exploratory microbiota studies face the challenge of determining whether the bacterial presence influences clinical outcomes or vice versa. To investigate this, we conducted an *in vitro* assay using a BC cell line similar to those from the KELLY trial: (HR[+]/HER2[-]). Since microbial metabolites are more likely to reach distant sites, such as tumors, via the bloodstream under physiological conditions, we incubated *B. fragilis* NCTC 9343 cell-free supernatants (CFS) from different growth phases with MCF-7 cells.

**Figure 6:**
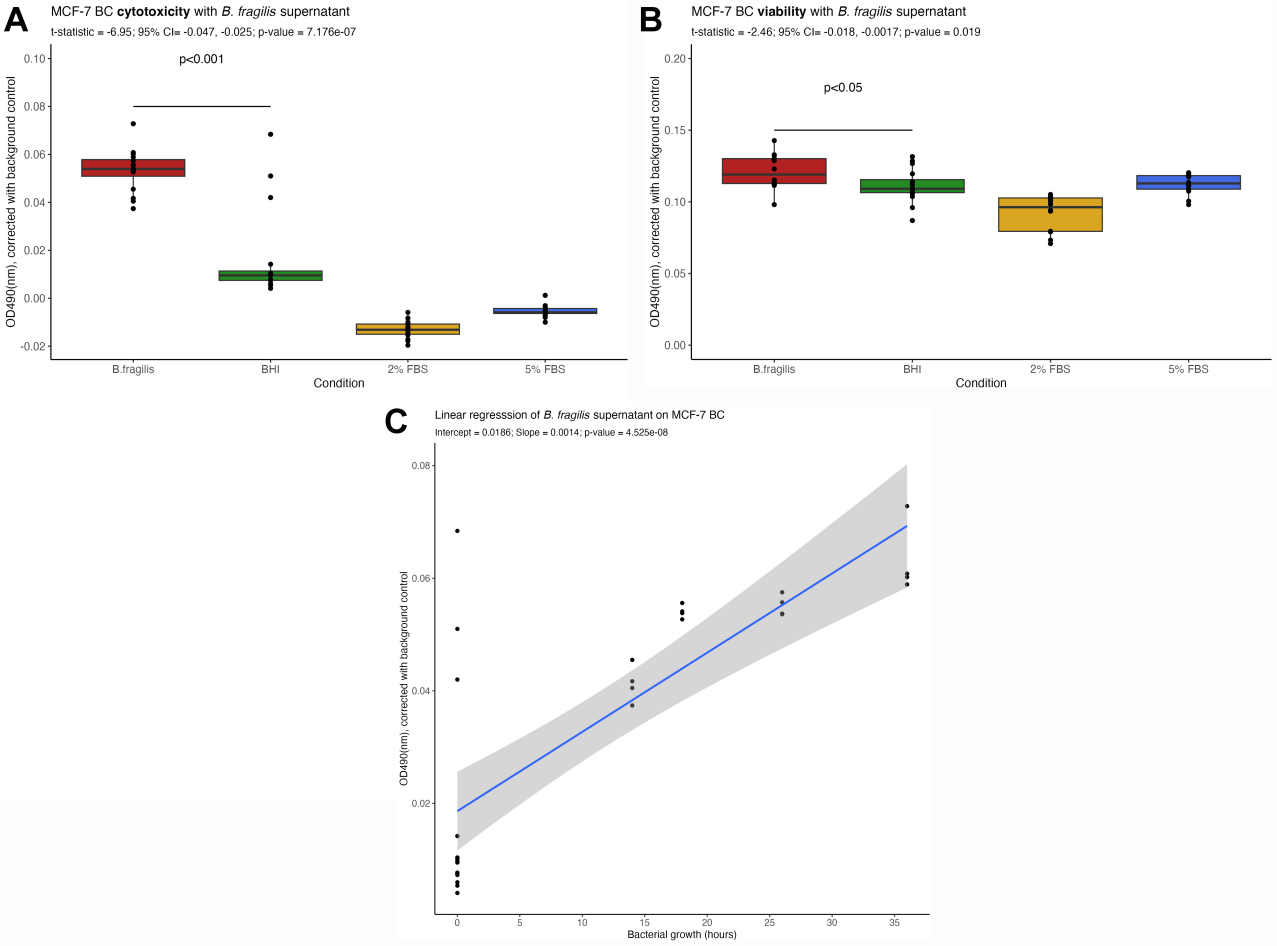
*B. fragilis* NCTC 9343 supernatant harvested during growth (0-36 h) could significantly (^***^P < 0.001) stimulate more LDH release, measured by OD_490_, from MCF-7 (HR[+]/HER2[-]) breast cancer cells *in vitro* compared to BHI control (A). However, this was not reflected as cell death since viability measured by OD_490_ (B) was not significantly different than BHI (^*^P < 0.05). Linear regression analysis showed that this was a dose dependent relationship (slope = 0.0186, *P*-value = 4.52 × 10^-8^%) (C). All conditions had *n* = 16, OD490 readings are corrected for background noise i.e., BHI media. Welch’s two sample *t*-test was used to compare the means of BHI and *B. fragilis* for both (A) and (B). LDH: Lactate dehydrogenase; BHI: brain-heart infusion.

Results showed that MCF-7 exposed to *B. fragilis* supernatant (from 14 to 36 h growth phases) released significantly more intracellular LDH compared to cells treated with BHI media alone [Figure 6A]. However, this did not lead to direct cell death [Figure 6B]. Instead, a significant reduction in metabolic activity (*P*-value = 0.019) was observed in MCF-7 cells exposed to *B. fragilis* supernatant, indicating compromised cell viability. Additionally, a linear regression analysis confirmed a significant and positive (slope = 0.0186, *P*-value = 4.52 × 10^-6^%), dose-dependent relationship between *B. fragilis* growth and cytotoxicity [Figure 6C].

## DISCUSSION

The gut and oral microbiota play key roles in human health, and recent research suggests these microbial communities may influence anti-cancer responses^[6-8,10-14,50-55]^. While most studies on the microbiota-cancer relationship have focused on melanoma or non-small cell lung cancer^[15,56,57]^, the CALADRIO study is one of the first to investigate the role of both gut and oral microbiota in BC patients undergoing novel combination therapy for mBC. Given the novelty of this therapeutic approach, we had a unique opportunity to comprehensively assess how this combination treatment influences both the oral and gut microbiota in mBC patients.

Patients who provided clinical samples for the CALADRIO study were enrolled in Spain, where previous work has identified a “typical” Spanish gut microbiota, including *Bacteroides*, *Faecalibacterium*, *Prevotella*, *Alistipes*, and *Oscillospiraceae*^[58]^. In agreement with these findings, we observed these core genera in the majority of our BC patient fecal samples [Figure 1A], with *Prevotella* also present in the oral microbiota samples at relatively high abundances. This suggests a potential oral-gut axis, as 57 genera appeared to be “common” between these two niches among patients. However, some taxa, such as *Capnocytophaga*, are more typically associated with the oral cavity than the gut [Supplementary Table 1]. Due to taxonomic resolution limitations with 16S rRNA gene amplicon sequencing, further exploration of the oral-gut axis was not possible. In terms of clinical parameters, several oral-associated microbes linked to the no-CB group were also detected in the gut samples after LEfSe analysis. These include *Streptococcus*_sp_KS_6, *Neisseria meningitidis*, and *Capnocytophaga sputigena* (though the latter result was likely influenced by two samples with high abundances, Supplementary Figure 3). This raises the possibility of translocation between body sites, which is relevant because oral microbes entering the gut have been implicated in diseases such as Crohn’s and colorectal cancer^[17]^. Notably, *Fusobacterium*, an oral-associated genus linked with colorectal cancer and BC in murine models, is not significant in our analysis^[59-62]^.

Unlike studies on melanoma, where higher gut diversity has been associated with a stronger response in immunotherapy^[11]^, we did not observe this in our cohort. This may be due to the nature of BC, which is not as immunogenic as melanoma, potentially resulting in a different microbiota host. Additionally, the patients in our cohort have undergone two rounds of prior chemotherapy, which could have altered their “BL” profiles.

Taxonomic shifts were observed primarily in the oral microbiota, particularly *Streptococcus*, which was more abundant in BL samples from CB patients, although the difference was not statistically significant. While *Streptococcus* is a common, and typically benign member of the oral microbiota^[63]^, it has been linked to cancers associated with the oral respiratory tract, such as oesophageal^[64]^, tongue^[65]^, and gastric cancer^[66]^. Despite these associations, the underlying mechanisms remain unclear. Interestingly, we observed a potential association between oral *Streptococcus* and CB at BL, though this trend disappeared by C4D1 and EoT, possibly due to antibiotic use, as all patients have received at least two different antibiotic classes by C4D1. Streptococci, such as *Streptococcus thermophilus*^[67]^ and *Streptococcus salivarius*, have been reported to have immune-modulatory effects^[68]^. However, the limitations of 16S rRNA gene amplicon sequencing hinder accurate species-level identification and the investigation of antimicrobial resistance profiles that could provide insights into the loss of this trend over time.

With respect to gut microbiota profiles, the observed association between *B. fragilis* and CB, although only significant at EoT, suggests that this species may serve as a potential biomarker for CB in the context of this novel therapy. However, a key challenge in exploratory microbiota studies is reliance on post-hoc analysis of sequencing data. Studies such as the one by Nearing *et al.* have highlighted the limitations of commonly used multi-variate analysis tools^[69]^, which can lead to false positives. Therefore, *in vitro* validation is needed to further investigate the validity of findings and establish possible causation. In our study, we conducted *in vitro* experiments and showed that a product produced by *B. fragilis* (using a type strain almost identical to those strains found in CB patients - as identified through MAGs) induced LDH release, indicating cellular stress, in MCF-7 BC cells. However, this effect was not linked to cell death, as cells retained metabolic activity, suggesting that the underlying mechanism by which gut microbiota changes influence BC outcomes remains unclear. It is plausible that gut microbiota alterations affect serum metabolites in cancer patients. A previous study, which modeled serum metabolites based on gut microbiota changes, identified eight gut-associated serum metabolites. While the study relied on *in silico* modeling, it lays the groundwork for further *in vitro* validation^[70]^. Additionally, other research has demonstrated that microbiota-derived metabolites, such as tryptophan, can influence tumor-associated macrophages and modulate antitumor activity, as shown in pancreatic ductal adenocarcinoma^[71]^. Although these studies have limitations, they provide evidence that gut microbiota changes can affect distal sites via metabolite production.

Vétizou *et al*. demonstrated the favorable outgrowth of *B. fragilis* in CTLA-4 anti-cancer blockade in RET-mutated melanoma and MC38 colon cancer models. Their study suggested that this effect may be achieved through TH1-mediated immune responses^[14]^, potentially linked to polysaccharide components of *B. fragilis*. Specifically, polysaccharide A (PSA), which is present on the surface of *B. fragilis* NCTC 9343, has been shown to promote mucosal immunity. In our study, 3 out of the 5 MAGs extracted from CB patients encoded these polysaccharide components, which may have been released into the CFS due to natural bacterial death during growth^[72]^. Other potential microbial products in the supernatant include extracellular vesicles (EVs) or short-chain fatty acids (SCFAs). For example, EVs by *Bacteroides thetaiotaomicron* have been reported to influence host immune pathways in inflammatory bowel disease^[73]^. SCFAs, which are bacterial by-products, have long been recognized for their beneficial effects on the host. Butyrate, for instance, has shown anti-inflammatory properties in colitis^[74]^, while other metabolites such as cadaverine and lithoholic acid have been reported to inhibit BC proliferation^[75,76]^. Given this, it is likely that *B. fragilis* secretes immunomodulatory compounds into the supernatant. Future metabolomics studies will be essential to fully characterize the secreted products. Clinically, these secreted products may contribute to an unfavorable tumor microenvironment^[10]^ or stimulate anti-cancer immune responses^[7,14]^, potentially leading to a CB in patients.

Studies investigating the relationship between the microbiota and responses to anti-PD-1 therapy have provided evidence that certain members may influence antitumor responses. For instance, Gopalakrishnan *et al*. assessed the oral and gut microbiota of melanoma patients receiving anti-PD-1 immunotherapy, finding that *Faecalibacterium* was linked to prolonged PFS and was significantly more abundant in responders. Interestingly, they also observed an enrichment of Bacteroidales in non-responders^[11]^. Similarly, Routy *et al*. reported an association between *Akkermansia muciniphila* and clinical responses mediated by interleukin-12^[13]^. From a treatment perspective, Tanoue *et al*. demonstrated that an eleven-strain microbial consortium, consisting of seven Bacteroidales strains and four non-Bacteroidales strains^[8]^, could prime CD 8 T cells for an antitumor response. However, no study to date has assessed the effect of eribulin on the gut microbiota, or any association with *Bacteroides* species and strains. While the results of these studies have been inconsistent, they do support the hypothesis that gut microbes can modulate the efficacy of anti-PD-1 therapy. The discrepancies between the studies may be due to variations in patient demographics and cancer types - this study focussed on BC, while others have examined non-small cell lung cancer and melanoma. Nevertheless, these findings underscore the importance of exploratory studies to investigate the potential of the microbiota as adjuvant options for enhancing immunotherapy outcomes.

While our investigation revealed promising insights into microbiota associations with clinical outcomes, it has several limitations. As this was an exploratory study, further well-powered multi-center studies comparing the gut and oral microbiota in a larger cohort of BC patients across treatment time points are necessary. Even when a microbiota association appears significant, the sample size can limit confidence in whether this is a genuine finding or a chance observation^[77]^. Thus, additional mechanistic work, *in vitro* and/or *in vivo*, could provide further evidence and potentially provide a platform for therapeutic development. Our *in vitro* analysis suggested that *B. fragilis* produces a metabolite or compound that stimulates LDH release from MCF-7 cells, although this effect did not reach cytotoxic levels. Since we only tested the total supernatant, further studies should aim to isolate and identify the specific compounds responsible for this activity. Moreover, the oral microbiota in our study was profiled using 16S rRNA gene amplicon sequencing, which has its limitations in resolution. Applying shotgun metagenomics profiling would allow for more detailed strain tracking and potential insights into oral-gut translocation. Finally, much of the current literature has focused on microbiota associations with immunotherapy efficacy, but there is limited information on how chemotherapy affects the microbiota, which may have influenced our findings^[78]^. Addressing these gaps in future research will help clarify the role of microbiota in cancer therapies.

We have demonstrated that the combination therapy of pembrolizumab and eribulin does not induce microbiota toxicity in Spanish pre-treated patients with HR[+]/HER2[-] mBC. The study is among the first to explore the impact of this novel combination on the oral and gut microbiota of mBC patients. Our findings suggest that BL oral *Streptococcus* may serve as a potential biomarker for CB, while gut-derived *B. fragilis* also appears to be associated with CB at EoT. *In vitro* studies indicated that *B. fragilis* may produce a product that affects MCF-7 BC cells, although it is not strongly cytotoxic. Further mechanistic studies are needed to elucidate the pathways involved, but a deeper understanding of *B. fragilis* and its secreted components may open up opportunities for developing therapeutic adjuvants in anti-cancer therapies.

## DECLARATIONS

### Availability of data and materials

The gut metagenome shotgun sequencing data can be found at PRJNA1050903 and the saliva 16S rRNA gene amplicon sequencing data can be found at PRJNA1052073. Code for the analysis undertaken in R can be found at: https://github.com/nteng22/CALADRIO-paper.

### Copyright

© The Author(s) 2024.
