# Supplementary material for: Profiling the gut and oral microbiota of hormone receptor-positive, HER2-negative metastatic breast cancer patients receiving pembrolizumab and eribulin
Source: Microbiome Res Rep. 2024 Oct 31;4(1):4. doi: 10.20517/mrr.2024.49 (PMC11977384; doi:10.20517/mrr.2024.49)
Supplement: Supplementary file 1 [file mrr-4-1-4-SupplementaryMaterials.pdf]

Supplementary Materials

Profiling the gut and oral microbiota of hormone receptor-positive, HER2-negative metastatic breast cancer patients receiving pembrolizumab and eribulin

Nancy MY Teng[1], Andrea Malfettone[2], Matthew J Dalby[3], Raymond Kiu[1,3], David Seki[4], Tim Robinson[5], María Gion[6], Begoña Bermejo[7], José Manuel Pérez-García[2,8], Aleix Prat[9], Raúl Márquez Vázquez[10], Antonio Llombart-Cussac[2,11], Giuseppe Curigliano[12,13], Peter Schmid[14], Romualdo Barroso-Sousa[15], Mario Mancino[2], Eileen Shimizu[2], Jose Rodríguez-Morató[2], Leonardo Mina[2], Lindsay J Hall[1,3,4,16], Stephen D Robinson[1,17], Javier Cortés[2,8,18]

[1]Gut Microbes&Health, Quadram Institute Bioscience, Norwich NR4 7UQ, UK.

[2]Medica Scientia Innovation Research (MEDSIR), Barcelona 08005, Spain.

[3]Institute of Microbiology and Infection, University of Birmingham, Birmingham B12 2TT, UK.

[4]Intestinal Microbiome, School of Life Sciences, ZIEL - Institute for Food&Health Technical University of Munich, Freising D-80333, Germany.

[5]Bristol Medical School, University of Bristol, Bristol BS8 2BN, UK.

[6]Medical Onology Department, Hospital Universitario Ramón y Cajal, Madrid 28034, Spain.

[7]Medical Oncology, Hospital Clínico de Valencia, INCLIVA, CIBERONC, Medicine Department, Universidad de Valencia, Valencia 46010, Spain.

[8]International Breast Cancer Center (IBCC), Pangaea Oncology, Quirón Group, Barcelona 08017, Spain.

[9]Medical Oncology Department, Hospital Clínic y Provincial de Barcelona, Barcelona 08036, Spain.

[10]Servicio de Oncología Médica, MD Anderson Cancer Center, Madrid 28033, Spain.

[11]Department of medical oncology, Hospital Arnau de Vilanova, FISABIO, Valencia 46800, Spain.

[12]European Institute of Oncology, IRCCS, University of Milano, Milano 20141, Italy.

[13]Department of Oncology and Hemato-Oncology, University of Milano, Milano 20122, Italy.

[14]Barts Cancer Institute, Queen Mary University of London, London E1 4NS, UK.

[15]Oncology Center, Hospital Sirio-Libanes, Brasília-DF 70200-730, Brazil.
[16]Norwich Medical School, University of East Anglia, Norwich NR4 7TJ, UK.
[17]School of Biological Sciences, University of East Anglia, Norwich NR4 7TJ, UK.
[18]Department of Medicine, Faculty of Biomedical and Health Sciences, Universidad Europea de Madrid, Madrid 28670, Spain.

Correspondence to: Dr. Stephen D Robinson, Prof. Lindsay J Hall, Gut Microbes&Health, Quadram Institute Bioscience, Norwich Research Park, Norwich NR4 7UQ, UK. E-mail: stephen.robinson@quadram.ac.uk; lindsay.hall@quadram.ac.uk

A

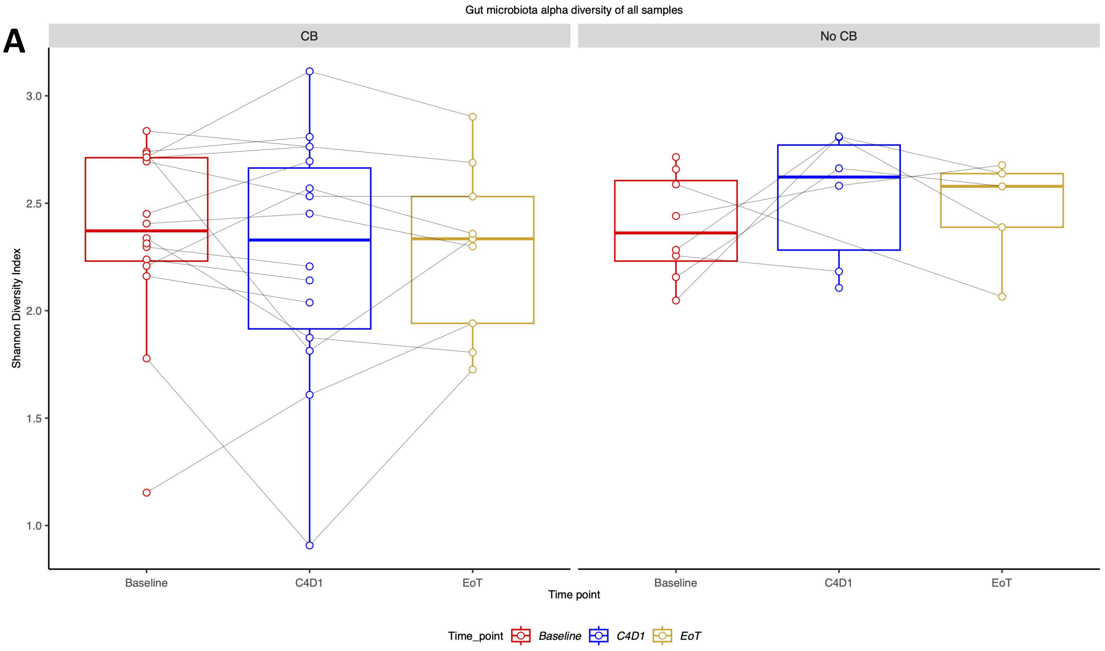

B

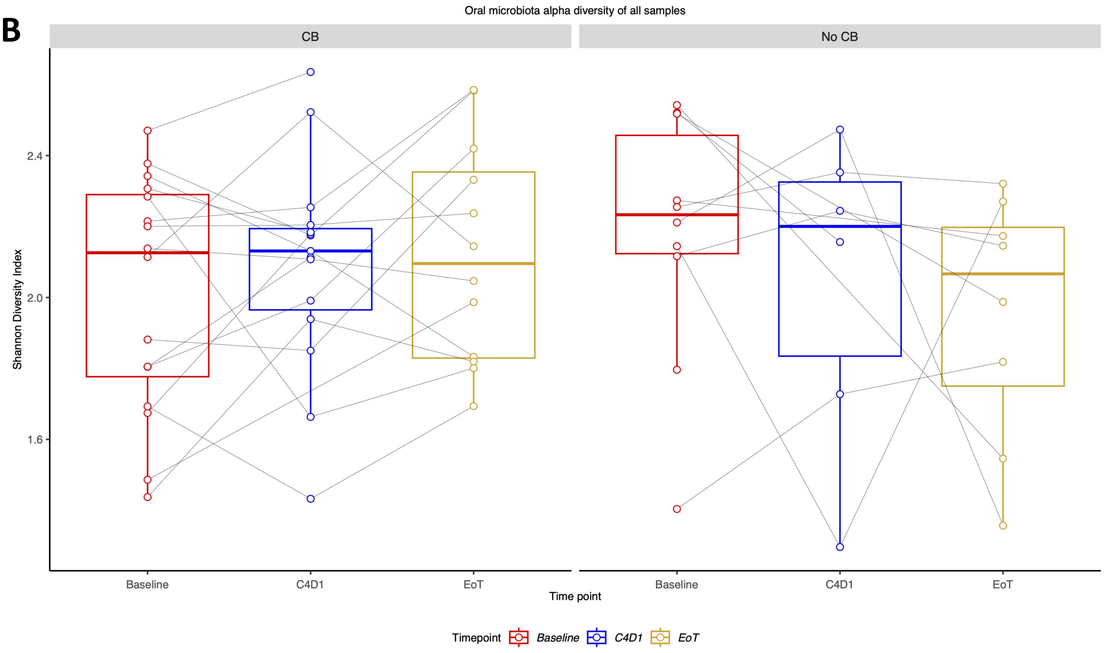

Supplementary Figure 1. Study treatment does not confer microbiota toxicity. No significant changes in the gut alpha diversity at the genus level were detected in patients who experienced a clinical benefit (CB) (A). Those patients who did not experience a CB showed a trend toward increasing gut alpha diversity, though not significant. This trend was not observed in the oral alpha diversity, also at the genus level (B). The line connecting the data points denotes samples from the same patient.

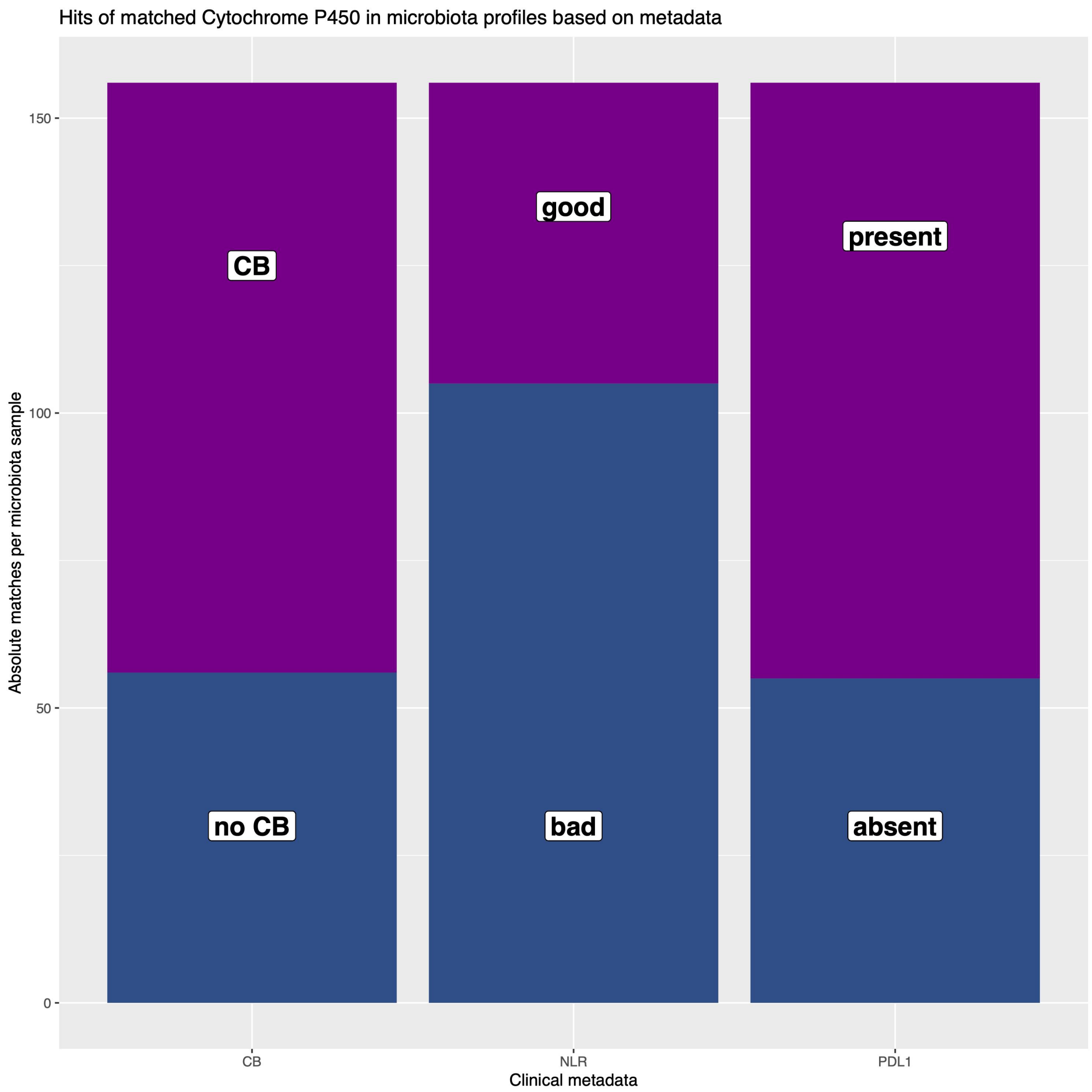

Supplementary Figure 2. Number of hits of Cytochrome P450 genes within each CALADRIO metagenome, stratified by clinical parameters: CB, NLR, PD-L1. CB: Clinical benefit; NLR: neutrophil-to-lymphocyte-ratio; PD-L1: programmed cell death ligand 1.

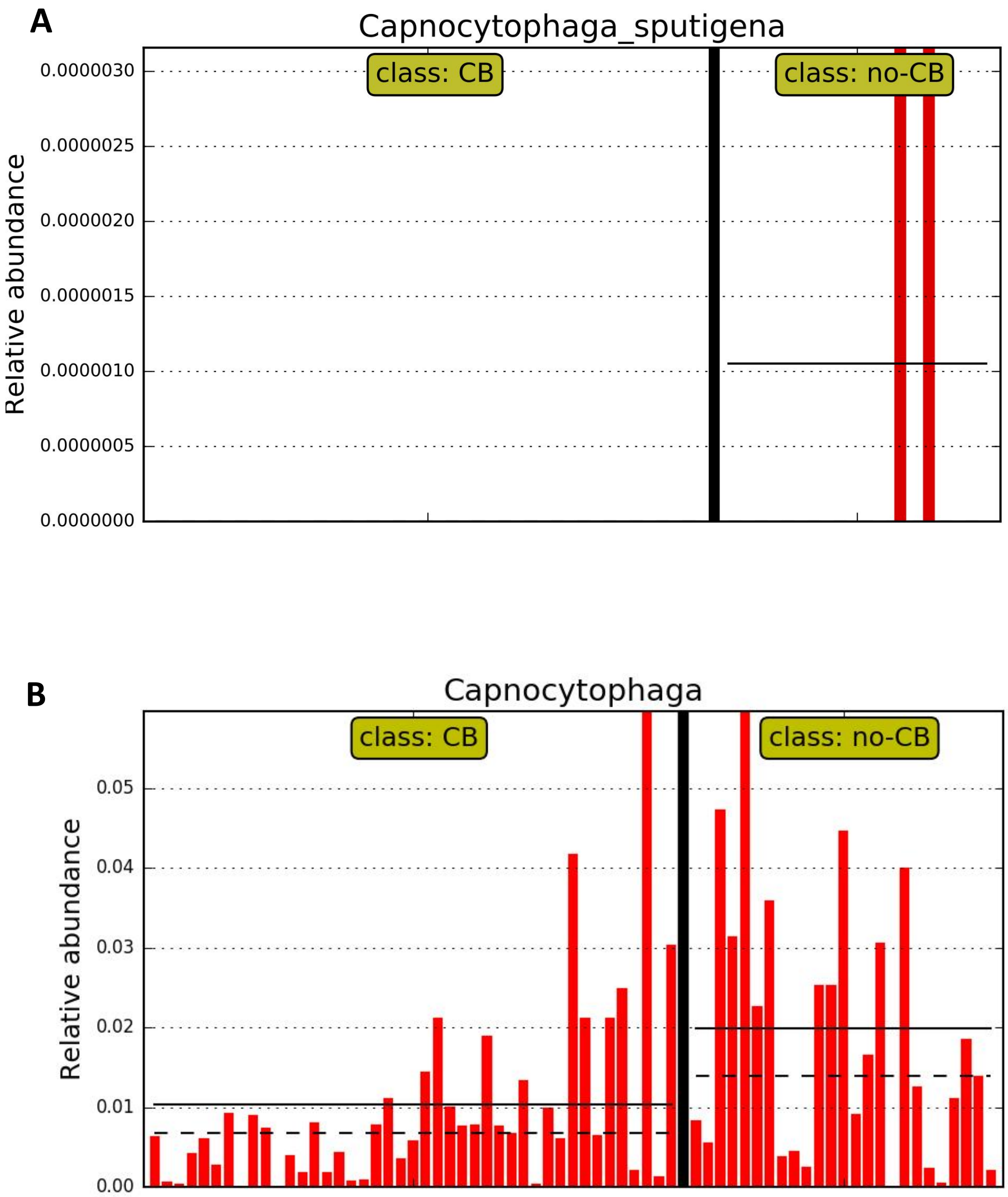

Supplementary Figure 3. Differential feature, Capnocytophaga, determined by LEfSe for (A) gut and (B) oral microbiota. Only two fecal samples had a high relative abundance of Capnocytophaga sputigena, suggesting it could be a false positive reported in LefSe analysis.

Supplementary Table 1. 57 common genera between the oral and gut microbiota

Acidovorax	*Desulfovibrio*	*Methylobacterium*
Acinetobacter	*Enterobacter*	*Mobiluncus*
Actinomyces	*Enterococcus*	*Mogibacterium*
Aggregatibacter	*Faecalibacterium*	*Mycoplasma*
Agrobacterium	*Filifactor*	*Neisseria*
Bacillus	*Flavobacterium*	*Odoribacter*
Bacteroides	*Fusobacterium*	*Paenibacillus*
Bifidobacterium	*Gemella*	*Paracoccus*
Blautia	*Geobacillus*	*Porphyromonas*
Brevibacillus	*Haemophilus*	*Prevotella*
Bulleidia	*Klebsiella*	*Pseudomonas*
Butyrivibrio	*Lachnospira*	*Ruminococcus*
Campylobacter	*Lactobacillus*	*Shewanella*
Capnocytophaga	*Lactococcus*	*Sphingomonas*
Chryseobacterium	*Lautropia*	*Staphylococcus*
Clostridium	*Leptotrichia*	*Stenotrophomonas*
Coprococcus	*Leuconostoc*	*Streptococcus*
Desulfobulbus	*Macrococcus*	*Treponema*
Desulfomicrobium	*Methanobrevibacter*	*Weissella*
